# Immobilization of Cd Through Biosorption by *Bacillus altitudinis* C10-4 and Remediation of Cd-Contaminated Soil

**DOI:** 10.3390/microorganisms13081798

**Published:** 2025-08-01

**Authors:** Tianyu Gao, Chenlu Zhang, Xueqiang Hu, Tianqi Wang, Zhitang Lyu, Lei Sun

**Affiliations:** 1 College of Life Sciences, Institute of Life Sciences and Green Development, Hebei University, Baoding 071002, China; gaotianyu12138@163.com (T.G.); 18662804937@139.com (C.Z.); huxq000817@163.com (X.H.); 18031918331@163.com (T.W.); 2 Key Laboratory of Microbial Diversity Research and Application of Hebei Province, Baoding 071002, China; 3 Engineering Research Center of Microbial Breeding and Conservation, Hebei Province, Baoding 071002, China

**Keywords:** heavy-metal-resistant bacteria, *Bacillus altitudinis*, cadmium, biosorption, bioremediation

## Abstract

In this study, a highly cadmium (II)-resistant bacterium strain, C10-4, identified as *Bacillus altitudinis*, was isolated from a sediment sample collected from Baiyangdian Lake, China. The minimum inhibitory concentration (MIC) of Cd(II) for strain C10-4 was 1600 mg/L. Factors such as the contact time, pH, Cd(II) concentration, and biomass dosage affected the adsorption of Cd(II) by strain C10-4. The adsorption process fit well to the Langmuir adsorption isotherm model and the pseudo-second-order kinetics model, based on the Cd(II) adsorption data obtained from the cells of strain C10-4. This suggests that Cd(II) is adsorbed by strain C10-4 cells via a single-layer homogeneous chemical adsorption process. According to the Langmuir model, the maximum biosorption capacity was 3.31 mg/g for fresh-strain C10-4 biomass. Cd(II) was shown to adhere to the bacterial cell wall through SEM-EDS analysis. FTIR spectroscopy further indicated that the main functional sites for the binding of Cd(II) ions on the cell surface of strain C10-4 were functional groups such as N-H, -OH, -CH-, C=O, C-O, P=O, sulfate, and phosphate. After the inoculation of strain C10-4 into Cd(II)-contaminated soils, there was a significant reduction (*p* < 0.01) in the exchangeable fraction of Cd and an increase (*p* < 0.01) in the sum of the reducible, oxidizable, and residual fractions of Cd. The results show that *Bacillus altitudinis* C10-4 has good potential for use in the remediation of Cd(II)-contaminated soils.

## 1. Introduction

With the rapid development of industrialization, urbanization, and intensive agriculture, heavy metal pollution in the soil has become a more serious problem. In China, cadmium (Cd) was the primary pollutant in agricultural soil, according to the China Ecological and Environmental Status Bulletin, for three consecutive years from 2019 to 2021 [1]. Cd has a wide range of applications in many industries, such as in nickel chromium batteries, plastics, glass, specialty coatings, pigments, and fluorescent quantum dots [2,3,4,5], which often lead to the contamination of water and soil with Cd. Cd is a kind of heavy metal that is not necessary for human health, is not part of the body’s structure or involved in metabolism [6] and is one of the Group 1 human carcinogens [7]. Due to its strong tendency toward environmental migration, Cd could accumulate in the soil and spread through the soil–plant system [8] and be transmitted to humans through the food chain. The accumulation of Cd in the human body has adverse effects on human health [9]. As a human carcinogen, Cd can cause brain, bone, and liver damage, lung inefficiency, renal dysfunction, and reproductive system and neurological disorders [10,11,12,13]. The potential ecological risk of Cd is expected to be twice as high in an optimistic scenario in 2026 as in the present conditions [14]. Therefore, removing cadmium contamination is essential.

There are various technological methods used in the remediation of Cd-contaminated soils through the immobilization of Cd [15,16,17]. In recent years, microbial remediation has attracted great attention due to its low cost, practicality, effectiveness, and environmental friendliness [18]. Through biosorption, precipitation, and immobilization, as well as migration facilitated by microbial electrolytic cells (MECs) and enhanced phytoremediation, microorganisms can effectively mitigate the effects of Cd contamination [19]. Due to the abundance of electronegative functional groups (hydroxyl, carboxyl, phosphate, and amino groups) on the cell surface under neutral and alkaline conditions, which can form strong bonds with Cd(II), biosorption is an essential method for the microbial remediation of Cd(II) pollution [19,20]. Cd-resistant bacteria have not only adapted to and survived in Cd-contaminated environments but also have the potential to reduce the bioavailable fractions of Cd and increase the stable fractions of Cd in contaminated soils [21,22]. Various bacterial genera (*Bacillus*, *Pseudomonas*, *Salmonella*, *Bifidobacterium*, *Serratia*, *Rhodobacter*, *Pantoea*, *Enterobacter*, *Kocuria*, *Stenotrophomonas*, *Micrococcus*, *Klebsiella,* etc.) from a variety of environments have been exploited for Cd bioremediation [13]. The genus *Bacillus* is one of the predominant bacterial genera in soil [23]. *Bacillus* spp. can form spores under extreme conditions to withstand significant environmental stresses, so they represent one of the best sustainable solutions for removing heavy metals from various environments, especially soil [24].

Some species of the genus *Bacillus*, such as *Bacillus cereus* [25], *Bacillus safensis* [26], *Bacillus subtilis* [27], *Bacillus proteolyticus* [28], *Bacillus paramycoides* [28], *Bacillus licheniformis* [29], *Bacillus megaterium* (currently, the correct name is *Priestia megaterium*) [30,31], *Bacillus altitudinis* [32] and *Bacillus siamensis* [33], have resistance to Cd and Cd adsorption potential. As of 15 January 2025, the genus *Bacillus* contained 111 species with correct names (LPSN, https://lpsn.dsmz.de/ accessed on 15 January 2025). Therefore, screening and identifying novel Cd-resistant bacteria and analyzing their characteristics can help improve the bioremediation process.

In this study, a Cd-resistant strain, C10-4, isolated from a sediment sample collected from Baiyangdian Lake in China, was identified as *Bacillus altitudinis*. The influence of various factors on the heavy metal adsorption capacity of the cells of strain C10-4 was investigated. Isotherm and kinetics models were used to evaluate the biosorption mechanism and process. The mechanism of the adsorption of the heavy metal Cd by strain C10-4 was further explored using scanning electron microscopy (SEM), energy-dispersive spectrometry (EDS), and Fourier infrared spectroscopy (FTIR). The distribution of the adsorbed heavy metal Cd in the bacterial cells was examined. The ability of strain C10-4 to remediate Cd-contaminated soil under the experimental conditions was also determined.

## 2. Materials and Methods

### 2.1. Minimum Inhibitory Concentration of Cd(II) and Multi-Metal Resistance

The Cd(II)-resistant bacterial strain C10-4 was isolated from a sediment sample collected from Yangzhuangzi (38°54′15″ N, 116°2′49″ E) in Baiyangdian Lake, China. A 1% inoculum of strain C10-4 (OD_600_ = 1) in the logarithmic growth period was inoculated into an LB medium with different concentrations of Cd(II), Pb(II), Zn(II), Cu(II), and Cr(VI). The cultures were incubated in an orbital shaker (180 rpm) at 25 °C for 48 h, and then the optical density at 600 nm (OD_600_) was recorded with a UV/Vis spectrophotometer (UV/Vis 1800, Mapada, Shanghai, China). The lowest heavy metal concentration that inhibited the growth of strain C10-4 was the minimum inhibitory concentration (MIC).

### 2.2. Bacterial Identification

The morphological characteristics of colonies of strain C10-4 were observed after being cultured on an LB agar plate at 25 °C for 18 h. According to the protocol outlined by Liu et al. [34], the cells were examined for their size and shape using a light microscope (ECLIPSE Ni-U, Nikon, Tokyo, Japan) and a scanning electron microscope (Regulus 8100, Hitachi, Tokyo, Japan) after being incubated on an LB agar plate for 18 h at 25 °C. Gram staining, endospore staining, and the biochemical characteristics, such as catalase activity, oxidase activity, Voges–Proskauer reaction, methyl red test, indole production, utilization of citrate and propionate, urease activity, and starch hydrolysis were tested using the methods described by Dong and Cai [35].

Genomic characterization and phylogenetic analysis based on the 16S rRNA gene, as well as the *gyrA*, *gyrB*, and *rpoB* gene sequences, were used to identify the genotype of strain C10-4. Genomic DNA was extracted using the genomic DNA extraction kit (Tiangen, Beijing, China). Subsequently, the draft-genome sequence of strain C10-4 was sequenced by the Illumina NovaSeq PE150 at Beijing Novogene Bioinformatics Technology Co., Ltd., Beijing, China. The genome assembly was carried out using the SOAP denovo software (version 2.04). Multi-locus sequence analysis (MLSA) was used to refine the taxonomic position of strain C10-4 [36]. Using MEGA X software [37], the 16S rRNA, *gyrA*, *gyrB*, and *rpoB* concatenated nucleotide sequences of strain C10-4 were aligned with the corresponding gene sequences of the related type strains. The nucleotide sequences of the 16S rRNA, *gyrA*, *gyrB*, and *rpoB* genes were obtained from the genomes of type strains of *Bacillus* species available at NCBI (www.ncbi.nlm.nih.gov/ accessed on 10 July 2024). The phylogenetic trees were reconstructed using the neighbor-joining method [38] according to Kimura’s two-parameter model [39] in the MEGA X program with 1000 bootstrap replicates [40]. Average nucleotide identity (ANI) was determined using EzBioCloud (https://www.ezbiocloud.net/tools/ani/ accessed on 10 July 2024) and digital DNA–DNA hybridization (dDDH) values were calculated using an online software tool (http://ggdc.dsmz.de/ accessed on 10 July 2024).

### 2.3. Preparation of the Cells of Strain C10-4

The strain C10-4 was harvested by centrifugation at 10,000× *g* for 10 min at 4 °C after growing to the exponential phase in LB medium under shaking conditions (180 rpm) at 25 °C. The cells were kept at 4 °C after being thoroughly cleaned three times with sterile deionized water before use.

### 2.4. Biosorption Experiments

#### 2.4.1. Effect of Physicochemical Parameters on Biosorption of Cd(II) by Strain C10-4

Some parameters, including contact time, pH, initial Cd(II) concentration, and biomass concentration, influenced the biosorption of Cd(II) by strain C10-4. First, the cells were reconstituted in an aqueous solution containing 40 mg/L Cd(II) (cadmium chloride solution) and incubated under the conditions of pH 6.0, 25 °C, 180 rpm to investigate the effect of contact time (15–300 min) for the adsorption of Cd(II) by strain C10-4, where the dosage of the cells was 6.0 g/L. Then, the effects of pH (4, 5, 6, 7, and 8), the initial concentration of Cd(II) (20–100 mg/L), and the biomass dosage (the cells 3.6–8.4 g/L) for the adsorption of Cd(II) by the strain C10-4 were conducted under similar conditions to those of the investigation into time, with the exception that the contact time was 60 min and the factors under investigation were set at different levels as needed. After incubation, the solution was centrifuged for 10 min at 10,000× *g*, 4 °C, and then filtered through microporous filter membranes with a pore size of 0.22 μm. The concentration of Cd(II) in the supernatant was measured with TAS-990 atomic absorption spectrophotometer (AAS) (Puxi General Instrument, China) after proper dilution. Each experiment was performed in triplicate. Equations (1) and (2) were used to determine the biosorption capacity and removal ratio of Cd(II):

(1) $$\begin{array}{c} q_{e}=\frac{\left(C_{i}-C_{e}\right)V}{m} \end{array}$$

(2) $$\begin{array}{c} R_{e}=\frac{C_{i}-C_{e}}{C_{i}}\times 100 \end{array}$$

where *q_e_* is the biosorption capacity in mg/g of biomass, *V* is the volume of Cd(II) solution (L), *C_i_* is the initial Cd(II) concentration in mg/L, *C_e_* is the Cd(II) concentration at equilibrium (mg/L), m is the biomass weight of strain C10-4 (g), and *R_e_* is the removal ratio of Cd(II) by percentage.

#### 2.4.2. Biosorption Isotherm and Kinetics

The relationship between the amount of metal ions per unit of bacterial biomass and the concentration of these metal ions in the solution is known as the biosorption isotherm [41]. The metal absorption capacity and the affinity between the metal and the biosorbent can be determined using the Langmuir and Freundlich isotherm models [42]. The adsorption rate and mechanism are described by pseudo-first order and pseudo-second order kinetics models in biosorption kinetics studies [43]. In the study, Langmuir and Freundlich isotherm models as well as pseudo-first and pseudo-second order kinetics models were used to analyze the biosorption data [42].

### 2.5. Characterization of Cd Immobilization by Strain C10-4

#### 2.5.1. SEM-EDS, FTIR Analysis

A scanning electron microscope (SEM) (Regulus 8100, HITACHI, Tokyo, Japan) and an energy-dispersive spectrometer (EDS) (AZtecLive Ultim Max 100, Oxford Instruments, Abingdon on Thames, UK) were used to monitor the surface properties of cells and the elemental distribution of heavy metal on cells of the C10-4 strain before and after Cd(II) adsorption [44]. The functional groups on the cell surface before and after Cd(II) treatment were analyzed using Fourier Transform Infrared Spectrometer (FTIR) (Nicolet IS5, Thermo, Waltham, MA, USA). In this process, the KBr matrix (Sigma, St. Louis, MO, USA) and the scanning wavelength in the range of 400–4000 cm^−1^ were used, at 4 cm^−1^ resolution.

#### 2.5.2. Bioaccumulation of Cadmium in Strain C10-4

The cells of strain C10-4 were suspended in 50 mL of the aqueous solution containing different concentrations of Cd(II) (20, 60, and 100 mg/L) and incubated for 1 h at 25 °C and 180 rpm, where the cell concentration was 6.0 g/L. Cells were harvested by centrifugation at 10,000× *g* for 10 min. To remove cadmium ions adsorbed on the cell surface, the cell pellets were resuspended with 20 mL of 100 mM EDTA, and incubated for 30 min at 30 °C and 160 rpm with shaking. The supernatant and cells were then separated by centrifugation at 10,000× *g* for 10 min. The supernatant was used to determine the extent of cell wall-bound Cd(II). To determine the amount of Cd(II) accumulated in the cells, the cell pellets were further digested with 5% HNO_3_ and 1% Triton X-100 for 30 min at 90 °C in a water bath. Cell lysate supernatant was then collected by centrifugation at 12,000× *g* for 10 min.

### 2.6. Bioremediation of Cd(II)-Contaminated Soils

#### 2.6.1. Preparation of Cd(II)-Contaminated Soils

Soil samples collected from the campus of Hebei University in China were dried at 60 °C and then passed through a 100-mesh sieve and divided into 9 aliquots. Cd(II) solution (1.0 g/L) was added to the soil samples and mixed thoroughly to prepare an artificially contaminated soil sample with initial Cd(II) concentrations of 50 and 100 mg/kg dry soil, respectively. Sterile deionized water was sprayed into the soil throughout the experiment to maintain a constant water-holding capacity of the soil (water-to-soil ratio 1:5). The process lasted 30 days. During this period, the soil was turned every 5 days and the moisture was maintained by weighing.

#### 2.6.2. Bioremediation and Analysis of Cd(II)-Contaminated Soil

Strain C10-4 was cultivated and harvested following the procedures outlined in Section 2.3. The collected cells were then resuspended in sterile deionized water, and adjusted to an OD_600_ of 1.2. The cell suspension was inoculated at 10% (*v*/*g*) into 500 g of Cd(II)-contaminated soil, while the same dose of sterile deionized water was inoculated into a control group. After an incubation period of 0, 5, 10, 15, 20, 25, and 30 days, soil samples (10 g) were collected from each treatment and stored in a refrigerator at −80 °C before use.

The different chemical fractions of cadmium in our soil samples were identified using the modified European Community Bureau of Reference (BCR) sequential extraction method [45], including acid soluble/exchangeable (F1), reducible (F2), oxidizable (F3), and residual fractions (F4). After each extraction procedure, the tubes were centrifuged at 4000× *g* for 20 min. Cd in the different extracts was measured by AAS in each of the extracted samples after filtering. Blank samples and the standard reference material were used for the quality control of the analysis.

### 2.7. Statistical Analysis

Three duplicates of each set of experiments were carried out. To ensure reproducibility, all experiments were repeated separately. All data analyses were performed using SPSS 23.0. With a significance level of *p* < 0.05, the data were evaluated using ANOVA and Tukey’s test.

## 3. Results and Discussion

### 3.1. MIC and Multi-Metal Resistance

The heavy-metal-resistance ability of strain C10-4 was evaluated on LB medium supplemented with different concentrations of heavy metal iron. Strain C10-4 showed high resistance to Cd(II) based on the results of the MIC test, up to 1600 mg/L. In addition, the multi-metal resistance capacity of strain C10-4 was evaluated, and it showed resistance to various toxic metals, including Pb(II) (200 mg/L), Cr(VI) (50 mg/L), Cu(II) (50 mg/L), and Zn(II) (25 mg/L). In contrast to previously documented cadmium-resistant bacteria such as *B. altitudinis* CdRPSD103 [32], *Burkholderia* sp. SRB-1 [46], *Pseudomonas* sp. strain 375 [47], *Escherichia coli* [48], and *Salmonella enterica* 43C [49], displaying maximum Cd(II) resistance values up to 1500 mg/L, 420 mg/L, (~672 mg/L) 6 mM, (~896 mg/L) 8 mM, and (~1500 mg/L) 13.3 mM, respectively, strain C10-4 showed significantly higher resistance to Cd(II).

### 3.2. Identification of Strain C10-4

**Figure 1:**
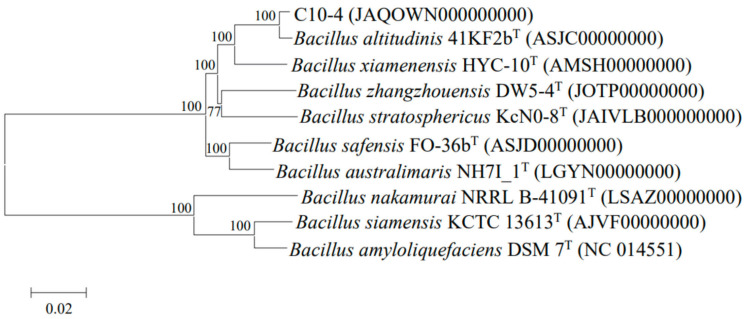
Neighbor-joining phylogenetic tree based on concatenated 16S rRNA, *gyrA*, *gyrB*, and *rpoB* gene sequences of strain C10-4 and related strain types of *Bacillus* species.

Strain C10-4 was a Gram-positive, non-motile, spore-forming rod-shaped bacterium, with a diameter of 0.5–1.2 µm and a length of 3.3–4.8 µm. After 18 h of incubation at 25 °C, the colonies were white, round, semitransparent, and slightly convex with whole edges, a glossy appearance, and a diameter of about 2 mm on LB agar plates. It was positive for catalase, oxidase, starch hydrolysis, and the methyl red test, and negative for indole production, the Voges–Proskauer test, urease activity, citrate use, and propionates. The nearly complete 16S rRNA gene sequence (1506 nt) of strain C10-4 was submitted to GenBank (accession number OQ345970) and aligned with the available sequences using Blast. Analysis of the 16S rRNA gene sequences revealed the highest sequence similarity to *Bacillus altitudinis* 41KF2bT (99.80%). Phylogenetic analysis of the 16S rRNA and the three housekeeping genes (*gyrA*, *gyrB*, and *rpoB*) showed that strain C10-4 was most closely related to *B. altitudinis* 41KF2bT (Figure 1). The draft-genome sequencing of strain C10-4 has been submitted to GenBank (accession number JAQOWN000000000) and is publicly available. Strain C10-4 had a genome of approximately 3.7 Mb, which included 3760 predicted genes as protein-coding sequences, 79 tRNA, and 10 rRNA gene copies, with a DNA G + C content of 41.32%. Strain C10-4 shared 84.7% of dDDH and 98.27% of ANI values with the strain type *B. altitudinis* 41KF2bT, which was higher than the proposed species boundary cut-off for dDDH and ANI (dDDH was 70% and ANI 95–96%) [50]. The results suggested that strain C10-4 should be identified as *Bacillus altitudinis*.

### 3.3. Biosorption of Cadmium by Strain C10-4

#### 3.3.1. Effect of Factors on Biosorption of Cd(II) by Strain C10-4

1. Effect of Contact Time

**Figure 2:**
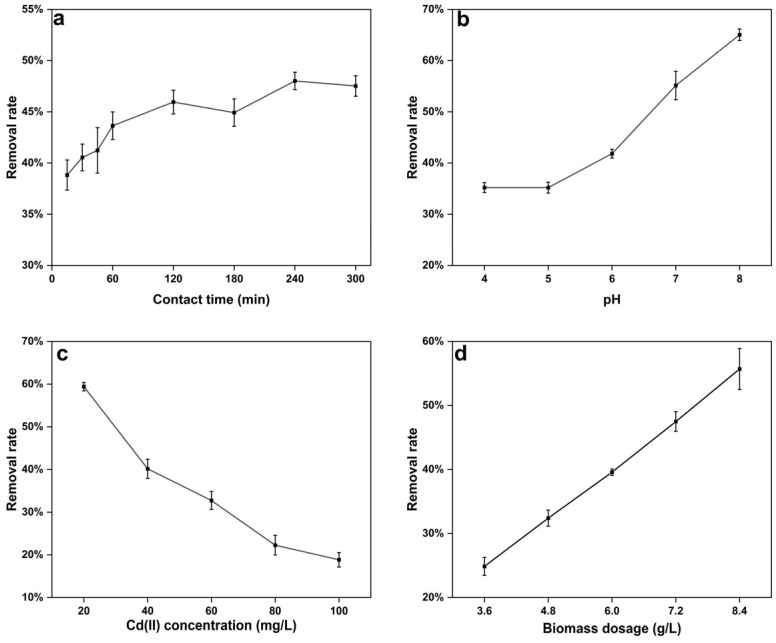
The effect of contact time (**a**), pH (**b**), cadmium concentration (**c**), and the biomass of the cells (**d**) on the biosorption of Cd by *Bacillus altitudinis* C10-4.

To evaluate the adsorption capacity of the cells of strain C10-4 on Cd(II), the removal ratios of Cd(II) of strain C10-4 in Cd solution at different contact times (0–300 min) were calculated (Figure 2a). The Cd(II) biosorption capacity of strain C10-4 increased steadily with increasing contact time from 15 min to 60 min. The removal rate of Cd(II) was close to the maximum value after 60 min, namely 43.64%, and the corresponding biosorption capacity was 2.91 mg/g. At 240 min, the maximum removal rate was 48.01%. Compared to the removal rate at 60 min, it only increased by about 4% at 240 min for strain C10-4. Metal ions can be adsorbed on the cell surface through electrostatic adsorption or complex interaction with various polar functional groups, resulting in a rapid process of metal adsorption by bacteria [16], and the adsorption reached equilibrium when all of the adsorption sites were bound. The time required for heavy metals in the solution to attain a constant value is known as the adsorption equilibrium time [51]. The adsorption equilibrium time indicates the sorption–desorption processes that occur after the saturation of the metal ions on the biomass surface [41]. The biosorption of Cd(II) by strain C10-4 was close to equilibrium at 60 min; this rapid equilibrium indicated that the biosorption process on the cell surface did not require an energy-mediated reaction [51]. In addition, the biosorption equilibrium time of Cd(II) by cadmium-resistant bacteria varied due to the fluctuations of biosorbents and the initial heavy metal concentrations [52].

2. Effect of pH

One of the most important factors influencing the biosorption of metal ions is the initial pH, which affects the surface functional groups of the adsorbent as well as the ionization process of the metal ions [44]. The influence of pH on the biosorption of Cd(II) by the cells of strain C10-4 at an initial Cd(II) concentration of 40 mg/L is shown in Figure 2b. The removal rate of Cd(II) of strain C10-4 increased steadily with increasing pH from 4.0 to 8.0, and the maximum ratio was 65.20% for pH values up to 8.0, corresponding to a biosorption capacity of 4.53 mg/g. In addition, the adsorption experiments were not performed on strain C10-4 with a pH above 8.0, because cadmium cations start to precipitate at pH values above 8.0. The results indicated that the pH of the solution affected the affinity of Cd(II) to negatively charged functional groups on the cell surface. Certain researchers like Mohapatra et al. [32], Hegazy et al. [51], Xu et al. [47], Khan et al. [49], Khadivinia et al. [53], and Masoudzadeh et al. [54] reported that the optimal pH of Cd(II) biosorption by *B. altitudinis* CdRPSD103, *Natronolimnobius innermongolicuswas*, *Pseudomonas* sp. 375, *S. enterica* 43C, *Ochrobactrum* sp. GDOS, and *Brevundimonas* sp. ZF12 were 7.0, 8.0, 7.0, 7.0, 6.0, and 8.0, respectively. Different cadmium-resistant bacteria have different optimal adsorption pHs, which mainly depend on the type of biosorbents (biomass) and sorbates (metal ions) used [55].

3. Effect of Cd(II) concentration

Using Cd(II) dosages ranging from 20 to 100 mg/L, the effect of initial Cd(II) concentration on biosorption was investigated (Figure 2c). The removal rate of Cd(II) decreased with increasing Cd(II) concentrations, while the corresponding biosorption capacity increased with increasing Cd(II) concentration. The cells of strain C10-4 showed an increase in biosorption capacities, from 1.98 mg/g to 3.14 mg/g. At a concentration of 80 mg/L Cd(II), the biosorption capacity of strain C10-4 approached saturation, which was mainly due to the saturation of the binding site of the adsorbent [43]. The results suggested that the biosorption capacity of strain C10-4 increased with the increase in the initial Cd concentration before the saturation of the binding site on the cell surface, which was consistent with other strains, such as *Bacillus* sp. B19, *B. altitudinis* CdRPSD103, and *N. innermongolicuswas* [32,51,56]. This may be because the higher concentration increases the chances of collisions between metal ions and adsorption sites [32]. However, the Cd(II) removal rate of strain C10-4 decreased with increasing cadmium concentration; the Cd(II) removal rates were 59.42% at a Cd(II) concentration of 20 mg/L and 18.85% at a Cd(II) concentration of 100 mg/L. This may be primarily attributed to the lack of sufficient free sites for additional Cd(II) ions to interact or combine with on the cell surface. Kailasam et al. [57] found that *Curtobacterium luteum* had the lower removal rate at a higher metal concentration due to the rapid saturation of binding sites at higher concentrations of metal ions.

4. Effect of biomass dosage

According to the results of some studies, the biomass dosage of the biosorbent and its functional groups play an essential role in determining the adsorption capacity and heavy metal removal rate [44,54,58]. The results of this study were fairly consistent with these. The removal rate of Cd(II) obviously increased with an increase in the biomass dosage of strain C10-4. When the biomass dosage of the cells was increased from 3.6 g/L to 8.4 g/L, the removal rate increased from 24.87% to 55.70% (Figure 2d). The adsorption capacity changed from 2.76 mg/g to 2.65 mg/g, and the difference was not significant (*p* > 0.05). It may be that under the conditions of this experiment the cadmium ion concentration is high enough that all adsorption sites on the cell surface of the adsorbent are occupied by metal ions.

#### 3.3.2. Modeling of Biosorption Isotherms and Kinetics

1. Modeling of biosorption isotherm

The adsorption isotherm models were used to evaluate the biosorption behavior and calculate the biosorption capacity. The Langmuir isotherm [59] assumes monolayer adsorption, and the formula can be given as follows:

(3) $$\begin{array}{c} \frac{C_{e}}{q_{e}}=\frac{1}{bq_{max}}+\frac{C_{e}}{q_{max}} \end{array}$$

where *q_e_* is the adsorbed amount of metal ions per gram sorbent (mg/g), *C_e_* is the adsorbate equilibrium concentration (mg/L), *q_max_* is the maximum metal adsorption capacity per unit weight biomass (mg/g), and b (L/mg) is the Langmuir constant.

The Freundlich isotherm [60] is an empirical equation based on sorption on a heterogeneous surface, and the formula can be given as follows:

(4) $$\begin{array}{c} lnq_{e}=lnk_{f}+\frac{1}{n}lnC_{e} \end{array}$$

where *q_e_* is the adsorbed amount of metal ions per gram sorbent (mg/g), *C_e_* is the adsorbate equilibrium concentration (mg/L), and *k_f_* and *n* are Freundlich constants (adsorption capacity and intensity, respectively).

**Figure 3:**
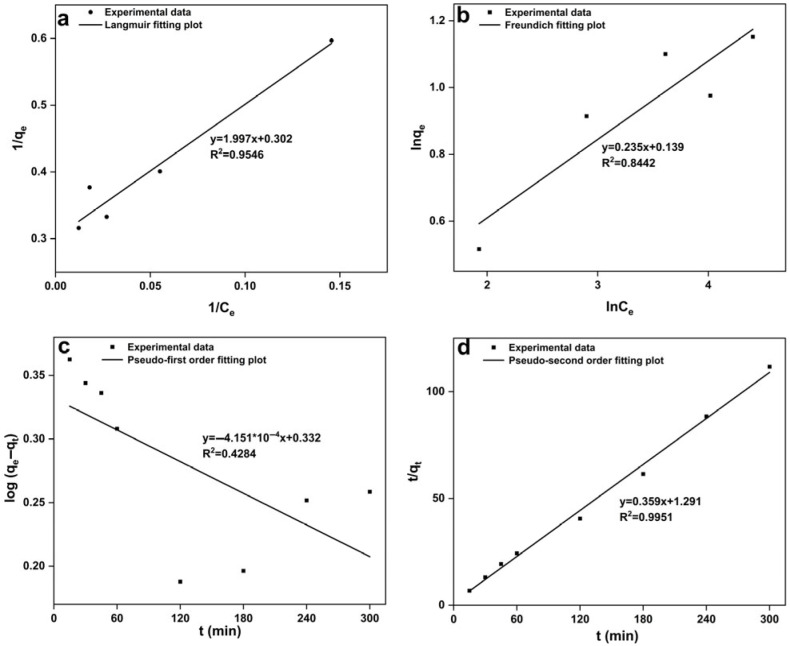
Isotherm model and kinetics model for Cd(II) biosorption by strain C10-4, (**a**) Langmuir isotherm model; (**b**) Freundlich isotherm model; (**c**) pseudo-first order kinetics; (**d**) pseudo-second order kinetics.

In this study, the data for Cd(II) adsorption by strain C10-4 at different concentrations were tested with Langmuir and Freundlich isotherm models (Figure 3a,b). The R^2^ values obtained from the Langmuir and Freundlich models were 0.9546 and 0.8442 for the cells of strain C10-4. The data showed that Cd(II) biosorption by C10-4 correlated better with the Langmuir model than with the Freundlich model. According to the Langmuir model, the maximum biosorption capacities were 3.31 mg/g for the fresh biomass of strain C10-4. The result suggests that Cd(II) biosorption by strain C10-4 belonged to monolayer adsorption. Similar to this, Cd(II) biosorption by other *Bacillus* sp., such as *Bacillus* sp. B19 [56], *Bacillus licheniformis* [61], *Bacillus laterosporus* MTCC 1628 [62], and *B. cereus* RC-1 [25], were found to be consistent with the Langmuir isotherm model.

2. Modeling of biosorption kinetics

Biosorption kinetics plays a crucial role in determining the feasibility and efficiency of biosorption processes [63]. In this experiment, through measuring the adsorption amount of Cd(II) by strain C10-4 at different adsorption times, the adsorption equilibrium data were fitted to the pseudo-first-order and pseudo-second-order kinetic models (Figure 3c,d). For the cells of strain C10-4, the pseudo-second-order kinetic model gave the R^2^ values up to 0.9951, it was higher than the R^2^ values of 0.4284 obtained with the pseudo-first-order kinetic model. The results indicated that Cd(II) biosorption by the cells of strain C10-4 correlated well with the pseudo-second-order kinetic model. Cd(II) biosorption by strain C10-4 occurred through the interaction of the surface functional groups and Cd(II) ion.

### 3.4. Mechanism of Cd Immobilization by Strain C10-4

#### 3.4.1. SEM and EDS Analysis

**Figure 4:**
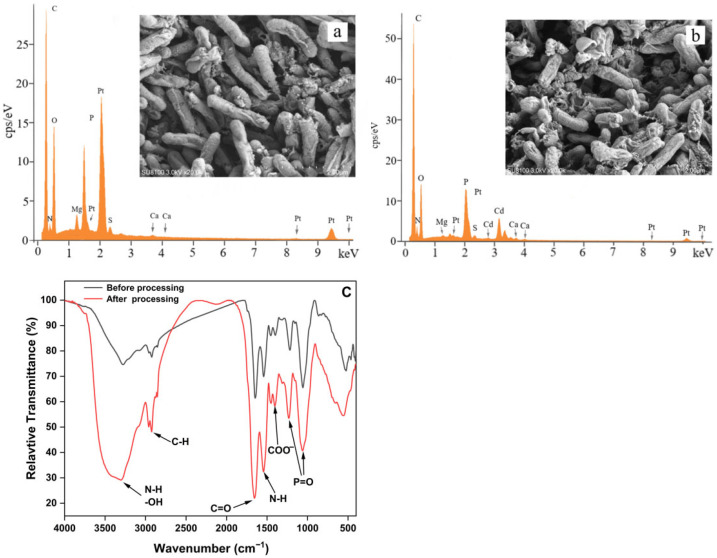
SEM and EDS images and FTIR analysis before and after the biosorption of Cd(II) by strain C10-4; (**a**) SEM and EDS images before the biosorption of Cd(II); (**b**) SEM and EDS images after the biosorption of Cd(II); (**c**) FTIR analysis.

Figure 4a,b shows SEM images and EDS analysis of the cells of *B. altitudinis* C10-4 treated with Cd(II) (40 mg/L) and without Cd(II) (control). The cells had a regular shape and the surface became rough after Cd biosorption. According to EDS analysis, Cd(II) adsorption dramatically changed the elemental composition of strain C10-4 cells. After Cd(II) treatment, high levels of cadmium were observed on the surface of the cells, with Cd metal detected as 3.76 wt.%. However, the control cells had no Cd. EDS analysis suggested that Cd(II) accumulated on the cell surface. Cell adsorption may be the main mechanism of Cd immobilization for strain C10-4 [64].

#### 3.4.2. FTIR Analysis

Figure 4c shows the FTIR spectral analysis of the change in absorption band assignment for different functional groups of strain C10-4 before and after interaction with Cd(II) in the range of 400–4000 cm^−1^. The characteristic infrared stretching frequencies of certain functional groups were used together with previous reports to identify the characteristic absorption bands of different functional groups [16,54,63,65,66,67]. After the cells of strain C10-4 interact with Cd(II), a band at 3304 cm^−1^ corresponds to the stretch bond of the N-H from amino group and indicates a bonded hydroxyl group [54]. Short peaks at 2924 cm^−1^ indicate stretching vibrations of -CH- in lipids [63]. The absorption bands corresponding to amide I (C=O) and amide II (N–H) in proteins were observed at 1655 cm^−1^, 1543 cm^−1^, indicating that the protein amide I band and II band on the cell wall of the strain C10-4 was active and participated in the immobilization of Cd(II) [68]. The absorption bands at 1403 cm^−1^ correspond to the symmetrical stretching vibration of the carboxylate ion (-COO-). The bands observed at 1231 cm^−1^ and 1061 cm^−1^ are attributed to P=O symmetry and P=O asymmetric elongation, respectively [67]. At the lower wave numbers (<580 cm^−1^), the peak shift shows that S- and P-containing groups contribute to the cellular absorption of Cd [54]. The shifts in these bands indicate the functional groups in cell surfaces of strain C10-4, such as carboxyl (-CO), hydroxyl (-OH), amine (-NH), phosphate and organic phosphate (P=O), and S-containing groups, have a direct influence on the immobilization of Cd(II) through the mechanisms of positive–negative charge binding, chelation, complexation, microprecipitation, and so on [68]. Upon comparing and analyzing the changes in the functional groups of *B. altitudinis* C10-4 and *B. altitudinis* CdRPSD103 after Cd treatment, it was found that the shifting of peak bands of the two strains was similar, indicating that the mechanisms of their adsorption of Cd were fundamentally identical [32].

#### 3.4.3. Distribution of Cd in Different Parts of Strain C10-4

**Figure 5:**
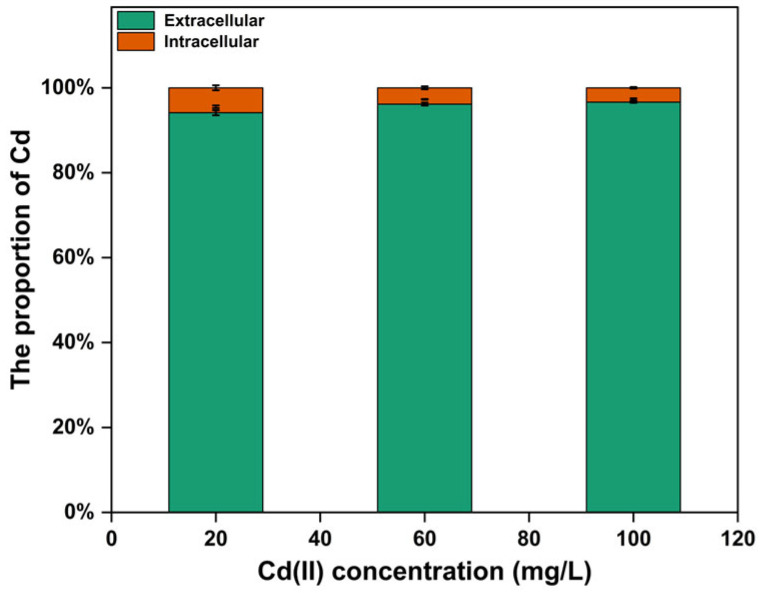
Cadmium on bacterial surface (biosorption) and inside cells (bioaccumulation) with different initial Cd(II) concentrations.

After 1 h of adsorption, the bioaccumulation of Cd(II) in the cells of strain C10-4 was examined at different initial Cd(II) concentrations (20, 60 and 100 mg/L) (Figure 5). The results showed that most Cd(II) were adsorbed on the cell surface and did not accumulate inside the cell. At a Cd(II) concentration of 20, 60, and 100 mg/L in the solution, the amount of Cd (II) adsorbed on the surface of the cells accounted for 94.12%, 96.16%, and 96.63% of the total removed cadmium ions, and the intracellular accumulation accounted for 5.88%, 3.84%, and 3.37%, respectively. Most of the Cd(II) in the solution was adsorbed by the functional groups on the cell surface, and only a small amount entered the cell. The results suggested that the Cd resistance of strain C10-4 was mainly due to the adsorption of Cd(II) on the cell surface, which reduced the accumulation of Cd(II) in the cell, and then prevented the toxic effect of Cd(II) on the cells. These results are similar to those of other researchers [25,29,56].

### 3.5. Analysis of the Bioremediation of Cd(II)-Contaminated Soil

**Figure 6:**
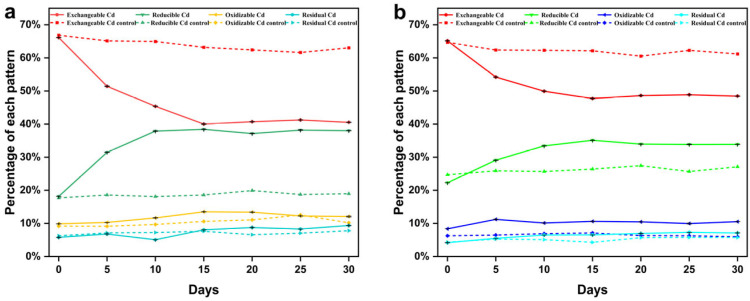
Remediation of contaminated soil with different initial Cd(II) concentrations of 50 mg/kg (**a**) or 100 mg/kg (**b**) by strain C10-4.

Cd can be divided into four fractions (exchangeable, reducible, oxidizable, and residue) by the BCR sequential extraction method. Normally, the exchangeable fraction of Cd is most likely to migrate into the environment and enter the food chain, while the reducible and oxidizable fractions of Cd are more inert than the exchangeable fraction of Cd, and the residual fraction of Cd is in a relatively stable state, as it has less biological toxicity to organisms [69]. The different cadmium chemical fractions in Cd(II)-contaminated soils treated with strain C10-4 were detected by the BCR. The percentages of cadmium from each extraction step are shown in Figure 6. According to the results, strains C10-4 had almost the same bioremediation effect on Cd(II) in soils containing 50 and 100 mg/kg cadmium. The exchangeable Cd gradually transformed into other Cd fractions, as evidenced by the significant decrease in its content and the increase in other fractions after bacterial inoculation. The exchangeable Cd decreased most rapidly within 0–5 days after the inoculation of strain C10-4. After that, the rate of decline gradually slowed until it reached stability after 15 days. On the 15th day, the exchangeable Cd in soils containing 50 and 100 mg/kg of cadmium decreased by 26.14% and 17.43%, and the reducible, oxidizable, and residual fractions Cd increased by 20.24%, 3.63%, 2.26%, and 12.83%, 2.26%, 2.35%, respectively. However, exchangeable Cd in the control groups (without bacteria inoculation) decreased by 3.65% and 2.54%, respectively. The sum of reducible, oxidizable, and residual Cd was significantly higher (*p* < 0.01) in the treatment group than in the control group. The different chemical fractions of cadmium varied in the control group without bacterial inoculation, which could be due to the activity of indigenous microorganisms [70]. In the study, the exchangeable fraction of Cd in Cd(II)-contaminated soils was significantly reduce (*p* < 0.01) and the sum of reducible, oxidizable, and residual Cd was significantly higher (*p* < 0.01) after the inoculation of strain C10-4, suggesting that strain C10-4 can reduce the bioavailability and liquidity of Cd in the soil, thereby immobilizing Cd(II) and remediating of Cd(II)-contaminated soil. Similar to strain C10-4, some strains can also reduce the bioavailability of Cd by immobilizing Cd, thereby reducing the harm of Cd in the soil. For example, compared with the control, the bioavailable Cd decreased by 53.47% in the soil containing 18.9 mg/kg cadmium inoculated with *Bacillus megaterium* A14 [31], the treatment with *Enterobacter* sp. LYX-2 reduced the content of available Cd in the soil (a Cd content of 1.26 mg/kg soil) by 15.92% [68], the bioavailable Cd content decreased by 53.79% in *Stenotrophomonas* sp. CD2-inoculated soil with a Cd content of 2 mg/kg [71]. Strain C10-4 reduced the bioavailable Cd in soils containing 50 mg/kg and 100 mg/kg cadmium by 22.49% and 14.89%, respectively. The microbial immobilization of Cd(II) has great potential in the bioremediation of heavy metals [72].

The immobilization of Cd in soil by microorganisms is affected by the physical and chemical properties of the soil, such as soil pH, soil organic matter, and other metal cations in the soil. Soil pH plays an important role in bioavailability, transformation processes, the distribution of heavy metals within the environment, and the protonation state of the functional groups on the microbial cell wall [20]. At high pH, the soil clay minerals’ surface charge may increase, leading to a shift from passive to specific sorption of Cd, which can reduce the bioavailability of Cd in the soil [73]. At low pH, some functional groups may be protonated, reducing their ability to bind Cd^2+^. Under pure culture conditions, the optimal pH for Cd biosorption by strain C10-4 was 8. Cations such as Ca^2+^ and Mg^2+^ in the soil will compete with Cd for binding sites on the surface of bacteria, reducing the immobilization efficiency of the strain on Cd in the soil. The presence of organic matter in the soil boosts its surface reactivity by interacting with heavy metal ions, such as Cd, thereby affecting the binding stability [31].

In this study, the remediation potential of strain C10-4 on Cd-contaminated soil was investigated under laboratory conditions. In order to apply strain C10-4 to the remediation of actual Cd-contaminated soils, further in-depth exploration is required in the following aspects: the long-term survival capability and Cd remediation activity of the strain, its impact on soil microorganisms, measures to avoid secondary pollution, the reuse of bioremediation agents, and the influencing and limiting factors for practical application.

## 4. Conclusions

In this study, *B. altitudinis* C10-4 was considered as a novel passivator with promising application prospects for the remediation of cadmium-contaminated environments. Strain C10-4 was capable of effectively removing the Cd(II) mainly through biosorption. The adsorption efficiency of the cells was related to contact time, pH, Cd(II) concentration, and biomass dosage. The experimental data obtained from isotherm and kinetics studies fit well with Langmuir isotherm and pseudo-second-order kinetic models. Studies involving SEM-EDS, FTIR, and the bioaccumulation of Cd(II) indicated that Cd(II) was mainly adsorbed on the cell wall by cell-surface functional groups. *B. altitudinis* C10-4 effectively reduced the bioavailability of cadmium pollutants in soil through the immobilization of Cd(II), and may have potential applications in the remediation of Cd-contaminated soil.

## Data Availability

The original contributions presented in this study are included in the article. Further inquiries can be directed to the corresponding authors.

## References

[1] The China Ecological and Environmental Status Bulletin. 2019–2021. https://www.mee.gov.cn/hjzl/sthjzk/zghjzkgb/.

[2] Huttunen-Saarivirta E., Korpiniemi H., Kuokkala V.T., Paajanen H. (2013). Corrosion of cadmium plating by runway de-icing chemicals: Study of surface phenomena and comparison of corrosion tests. Surf. Coat. Technol..

[3] Mo D., Hu L., Zeng G., Chen G., Wan J., Yu Z., Huang Z., He K., Zhang C., Cheng M. (2017). Cadmium-containing quantum dots: Properties, applications, and toxicity. Appl. Microbiol. Biotechnol..

[4] Turner A. (2019). Cadmium pigments in consumer products and their health risks. Sci. Total Environ..

[5] Guo J., Luo X., Zhang Q., Duan X., Yuan Y., Zheng S. (2024). Contributions of selenium-oxidizing bacteria to selenium biofortification and cadmium bioremediation in a native seleniferous Cd-polluted sandy loam soil. Ecotoxicol. Environ. Saf..

[6] Liu K., Li F., Cui J., Yang S., Fang L. (2020). Simultaneous removal of Cd(II) and As(III) by graphene-like biochar-supported zero-valent iron from irrigation waters under aerobic conditions: Synergistic effects and mechanisms. J. Hazard. Mater..

[7] IARC (2018). Agents Classified by the IARC Monographs. https://monographs.iarc.fr/agents-classified-by-the-iarc.

[8] Shi G.L., Zhu S., Bai S.N., Xia Y., Lou L.Q., Cai Q.S. (2015). The transportation and accumulation of arsenic, cadmium, and phosphorus in 12 wheat cultivars and their relationships with each other. J. Hazard. Mater..

[9] Anetor J.I. (2012). Rising environmental cadmium levels in developing countries: Threat to genome stability and health. Niger. J. Physiol. Sci..

[10] Rani A., Kumar A., Lal A., Pant M. (2014). Cellular mechanisms of cadmium-induced toxicity: A review. Int. J. Environ. Health Res..

[11] Rinaldi M., Micali A., Marini H., Adamo E.B., Puzzolo D., Pisani A., Trichilo V., Altavilla D., Squadrito F., Minutoli L. (2017). Cadmium, organ toxicity and therapeutic approaches: A review on brain, kidney and testis damage. Curr. Med. Chem..

[12] Cabral Pinto M.M.S., Ordens C.M., Condesso de Melo M.T., Inácio M., Almeida A., Pinto E., Ferreira da Silva E.A. (2020). An inter-disciplinary approach to evaluate human health risks due to long-term exposure to contaminated groundwater near a chemical complex. Expo. Health.

[13] Kumar A., Subrahmanyam G., Mondal R., Cabral-Pinto M.M.S., Shabnam A.A., Jigyasu D.K., Malyan S.K., Fagodiya R.K., Khan S.A., Kumar A. (2021). Bio-remediation approaches for alleviation of cadmium contamination in natural resources. Chemosphere.

[14] He M., Yan P., Yu H., Yang S., Xu J., Liu X. (2020). Spatiotemporal modeling of soil heavy metals and early warnings from scenarios-based prediction. Chemosphere.

[15] Kurniawan T.A., Chan G.Y., Lo W.H., Babel S. (2006). Physico–chemical treatment techniques for wastewater laden with heavy metals. Chem. Eng. J..

[16] Li J., Liu Y.R., Zhang L.M., He J.Z. (2019). Sorption mechanism and distribution of cadmium by different microbial species. J. Environ. Manag..

[17] Huang C., Guo Z., Peng C., Anaman R., Zhang P. (2023). Immobilization of Cd in the soil of mining areas by Fe-Mn oxidizing bacteria. Sci. Total Environ..

[18] Zhao X., Teng Z., Wang G., Luo W., Guo Y., Ji X., Hu W., Li M. (2023). Anaerobic syntrophic system composed of phosphate solubilizing bacteria and dissimilatory iron reducing bacteria induces cadmium immobilization via secondary mineralization. J. Hazard. Mater..

[19] Xia X., Wu S., Zhou Z., Wang G. (2021). Microbial Cd(II) and Cr(VI) resistance mechanisms and application in bioremediation. J. Hazard. Mater..

[20] Yan Z., Li Y., Peng S.Y., Wei L., Zhang B., Deng X.Y., Zhong M., Cheng X. (2024). Cadmium biosorption and mechanism investigation using two cadmium-tolerant microorganisms isolated from rhizosphere soil of rice. J. Hazard. Mater..

[21] Peng W., Li X., Song J., Jiang W., Liu Y., Fan W. (2018). Bioremediation of cadmium- and zinc-contaminated soil using *Rhodobacter sphaeroides*. Chemosphere.

[22] Han H., Wu X., Hui R., Xia X., Chen Z., Yao L., Yang J. (2022). Synergistic effects of Cd-loving *Bacillus* sp. N3 and iron oxides on immobilizing Cd and reducing wheat uptake of Cd. Environ. Pollut..

[23] Saxena A.K., Kumar M., Chakdar H., Anuroopa N., Bagyaraj D.J. (2020). *Bacillus* species in soil as a natural resource for plant health and nutrition. J. Appl. Microbiol..

[24] Wróbel M., Śliwakowski W., Kowalczyk P., Kramkowski K., Dobrzyński J. (2023). Bioremediation of heavy metals by the genus Bacillus. Int. J. Environ. Res. Public Health.

[25] Huang F., Dang Z., Guo C.L., Lu G.N., Gu R.R., Liu H.J., Zhang H. (2013). Biosorption of Cd(II) by live and dead cells of Bacillus cereus RC-1 isolated from cadmium-contaminated soil. Colloids Surf. B Biointerfaces.

[26] Nazli F., Jamil M., Hussain A., Hussain T. (2020). Exopolysaccharides and indole-3-acetic acid producing *Bacillus safensis* strain FN13 potential candidate for phytostabilization of heavy metals. Environ. Monit. Assess..

[27] Li W.L., Wang J.F., Lv Y., Dong H.J., Wang L.L., He T., Li Q.S. (2020). Improving cadmium mobilization by phosphate-solubilizing bacteria via regulating organic acids metabolism with potassium. Chemosphere.

[28] Saran A., Imperato V., Fernandez L., Gkorezis P., d’Haen J., Merini L.J., Vangronsveld J., Thijs S. (2020). Phytostabilization of polluted military soil supported by bioaugmentation with PGP-trace element tolerant bacteria isolated from Helianthus petiolaris. Agronomy.

[29] Liu H., Hong Z., Lin J., Huang D., Ma L.Q., Xu J., Dai Z. (2023). Bacterial coculture enhanced Cd sorption and as bioreduction in co-contaminated systems. J. Hazard. Mater..

[30] Gupta R.S., Patel S., Saini N., Chen S. (2020). Robust demarcation of 17 distinct *Bacillus* species clades, proposed as novel *Bacillaceae* genera, by phylogenomics and comparative genomic analyses: Description of *Robertmurraya kyonggiensis* sp. nov. and proposal for an emended genus Bacillus limiting it only to the members of the Subtilis and Cereus clades of species. Int. J. Syst. Evol. Microbiol..

[31] Yao X., Ren J., Fang L., Sun K., He W. (2024). The role and mechanism of Bacillus megaterium strain A14 in inhibiting the cadmium uptake by peanut plants in acidic red soil. J. Appl. Microbiol..

[32] Mohapatra R.K., Nayak M., Parhi P.K., Pandey S., Thatoi H., Panda C.R., Choi Y. (2024). Biosorption performance and mechanism insights of live and dead biomass of halophilic *Bacillus altitudinis* strain CdRPSD103 for removal of Cd(II) from aqueous solution. Int. Biodeterior. Biodegrad..

[33] Liu S., Huang Y., Zheng Q., Zhan M., Hu Z., Ji H., Zhu D., Zhao X. (2024). Cd-Resistant plant growth-promoting rhizobacteria *Bacillus siamensis* R27 absorbed Cd and reduced Cd accumulation in Lettuce (*Lactuca sativa* L.). Microorganisms.

[34] Liu Y., Zhai L., Yao S., Cao Y., Cao Y., Zhang X., Su J., Ge Y., Zhao R., Cheng C. (2015). *Brachybacterium hainanense* sp. nov., isolated from noni (*Morinda citrifolia* L.) branch. Int. J. Syst. Evol. Microbiol..

[35] Dong X.Z., Cai M.Y. (2001). Determinative Manual for Routine Bacteriology.

[36] Glaeser S.P., Kämpfer P. (2015). Multilocus sequence analysis (MLSA) in prokaryotic taxonomy. Syst. Appl. Microbiol..

[37] Kumar S., Stecher G., Li M., Knyaz C., Tamura K. (2018). MEGA X: Molecular evolutionary genetics analysis across computing platforms. Mol. Biol. Evol..

[38] Saitou N., Nei M. (1987). The neighbor-joining method: A new method for reconstructing phylogenetic trees. Mol. Biol. Evol..

[39] Kimura M. (1980). A simple method for estimating evolutionary rates of base substitutions through comparative studies of nucleotide sequences. J. Mol. Evol..

[40] Felsenstein J. (1985). Confidence limits on phylogenies: An approach using the bootstrap. Evolution.

[41] Aryal M., Liakopoulou-Kyriakides M. (2015). Bioremoval of heavy metals by bacterial biomass. Environ. Monit. Assess..

[42] Limcharoensuk T., Sooksawat N., Sumarnrote A., Awutpet T., Kruatrachue M., Pokethitiyook P., Auesukaree C. (2015). Bioaccumulation and biosorption of Cd^2+^ and Zn^2+^ by bacteria isolated from a zinc mine in Thailand. Ecotoxicol. Environ. Saf..

[43] Paul M.L., Samuel J., Chandrasekaran N., Mukherjee A. (2012). Comparative kinetics, equilibrium, thermodynamic and mechanistic studies on biosorption of hexavalent chromium by live and heat killed biomass of *Acinetobacter junii* VITSUKMW2, an indigenous chromite mine isolate. Chem. Eng. J..

[44] Mohapatra R.K., Parhi P.K., Pandey S., Bindhani B.K., Thatoi H., Panda C.R. (2019). Active and passive biosorption of Pb(II) using live and dead biomass of marine bacterium *Bacillus xiamenensis* PbRPSD202: Kinetics and isotherm studies. J. Environ. Manag..

[45] Cao L., Tian H., Yang J., Shi P., Lou Q., Ni Z., Peng X. (2015). Multivariate analyses and evaluation of heavy metals by chemometric BCR sequential extraction method in surface sediments from Lingdingyang Bay, South China. Sustainability.

[46] Liu H., Huang H., Liang K., Lin K., Shangguan Y., Xu H. (2023). Characterization of a cadmium-resistant functional bacteria (*Burkholderia* sp. SRB-1) and mechanism analysis at physiochemical and genetic level. Environ. Sci. Pollut. Res. Int..

[47] Xu S., Xing Y., Liu S., Hao X., Chen W., Huang Q. (2020). Characterization of Cd^2+^ biosorption by *Pseudomonas* sp. strain 375, a novel biosorbent isolated from soil polluted with heavy metals in Southern China. Chemosphere.

[48] Qin W., Zhao J., Yu X., Liu X., Chu X., Tian J., Wu N. (2019). Improving cadmium resistance in Escherichia coli through continuous genome evolution. Front. Microbiol..

[49] Khan Z., Rehman A., Hussain S.Z., Nisar M.A., Zulfiqar S., Shakoori A.R. (2016). Cadmium resistance and uptake by bacterium, *Salmonella enterica* 43C, isolated from industrial effluent. AMB Express.

[50] Chun J., Oren A., Ventosa A., Christensen H., Arahal D.R., da Costa M.S., Rooney A.P., Yi H., Xu X.W., De Meyer S. (2018). Proposed minimal standards for the use of genome data for the taxonomy of prokaryotes. Int. J. Syst. Evol. Microbiol..

[51] Hegazy G.E., Soliman N.A., Ossman M.E., Abdel-Fattah Y.R., Moawad M.N. (2023). Isotherm and kinetic studies of cadmium biosorption and its adsorption behaviour in multi-metals solution using dead and immobilized archaeal cells. Sci. Rep..

[52] Xia L., Xu X., Zhu W., Huang Q., Chen W. (2015). A comparative study on the biosorption of Cd^2+^ onto *Paecilomyces lilacinus* XLA and *Mucoromycote* sp. XLC. Int. J. Mol. Sci..

[53] Khadivinia E., Sharafi H., Hadi F., Zahiri H.S., Modiri S., Tohidi A., Mousavi A., Salmanian A.H., Noghabi K.A. (2014). Cadmium biosorption by a glyphosate-degrading bacterium, a novel biosorbent isolated from pesticide-contaminated agricultural soils. J. Ind. Eng. Chem..

[54] Masoudzadeh N., Zakeri F., Lotfabad T.B., Sharafi H., Masoomi F., Zahiri H.S., Ahmadian G., Noghabi K.A. (2011). Biosorption of cadmium by *Brevundimonas* sp. ZF12 strain, a novel biosorbent isolated from hot-spring waters in high background radiation areas. J. Hazard. Mater..

[55] Tunali S., Çabuk A., Akar T. (2006). Removal of lead and copper ions from aqueous solutions by bacterial strain isolated from soil. Chem. Eng. J..

[56] Liu S.Y., Pu Q., Mo T.J., Peng G., Sun Y., Zhang Y., Wang J., Li Y., Xu H.J. (2023). The mechanism of immobilization of Cd (II) by phosphate-solubilizing bacteria *Bacillus* sp. B19. Water Air Soil Pollut..

[57] Kailasam S., Sundaramanickam A., Kanth S.V. (2024). Isotherms and kinetics of multi-heavy metal sorption by marine phosphate-solubilizing bacteria from seagrass meadow. Int. J. Environ. Sci. Technol..

[58] Ren G., Jin Y., Zhang C., Gu H., Qu J. (2015). Characteristics of *Bacillus* sp. PZ-1 and its biosorption to Pb(II). Ecotoxicol. Environ. Saf..

[59] Langmuir I. (1918). The adsorption of gases on plane surfaces of glass, mica and platinum. J. Am. Chem. Soc..

[60] Freundlich H.M.F. (1906). Über die adsorption in losungen. Z. Phys. Chem..

[61] Baran M.F., Duz M.Z. (2019). Removal of cadmium (II) in the aqueous solutions by biosorption of *Bacillus licheniformis* isolated from soil in the area of Tigris River. Int. J. Environ. Anal. Chem..

[62] Kulkarni R.M., Shetty K.V., Srinikethan G. (2014). Cadmium (II) and nickel (II) biosorption by *Bacillus laterosporus* (MTCC 1628). J. Taiwan Inst. Chem. Eng..

[63] Rezaee A., Ahmady-Asbchin S. (2023). Removal of toxic metal Cd (II) by *Serratia bozhouensis* CdIW2 using in moving bed biofilm reactor (MBBR). J. Environ. Manag..

[64] Ma B., Wang J., Zhang L. (2023). Two cadmium-resistant strains of agricultural soil effective in remediating soil cadmium pollution. J. Environ. Chem. Eng..

[65] Deepika K.V., Raghuram M., Kariali E., Bramhachari P.V. (2016). Biological responses of symbiotic Rhizobium radiobacter strain VBCK1062 to the arsenic contaminated rhizosphere soils of mung bean. Ecotoxicol. Environ. Saf..

[66] Huang H., Jia Q., Jing W., Dahms H.U., Wang L. (2020). Screening strains for microbial biosorption technology of cadmium. Chemosphere.

[67] Lu C.W., Ho H.C., Yao C.L., Tseng T.Y., Kao C.M., Chen S.C. (2023). Bioremediation potential of cadmium by recombinant *Escherichia coli* surface expressing metallothionein MTT5 from Tetrahymena thermophila. Chemosphere.

[68] Luo Y., Liao M., Lu X., Xu N., Xie X., Gao W. (2024). Unveiling the performance of a novel alkalizing bacterium *Enterobacter* sp. LYX-2 in immobilization of available Cd. J. Environ. Sci..

[69] Cui J., Wang W., Peng Y., Zhou F., He D., Wang J., Chang Y., Yang J., Zhou J., Wang W. (2019). Effects of simulated Cd depositon on soil Cd availability, microbial response, and crop cd uptake in the passivation-remediation process of Cd-contaminated purple soil. Sci. Total Environ..

[70] Wang Y., Luo Y., Zeng G., Wu X., Wu B., Li X., Xu H. (2020). Characteristics and in situ remediation effects of heavy metal immobilizing bacteria on cadmium and nickel co-contaminated soil. Ecotoxicol. Environ. Saf..

[71] Fan X., Yuan K., Peng Q., Lv R., Zheng Y. (2024). *Stenotrophomonas* strain CD2 reduces cadmium accumulation in *Brassica rapa* L.. Front. Sustain. Food Syst..

[72] Wu S., Zhou Z., Zhu L., Zhong L., Dong Y., Wang G., Shi K. (2022). Cd immobilization mechanisms in a *Pseudomonas* strain and its application in soil Cd remediation. J. Hazard. Mater..

[73] Zeng F., Ali S., Zhang H., Ouyang Y., Qiu B., Wu F., Zhang G. (2011). The influence of pH and organic matter content in paddy soil on heavy metal availability and their uptake by rice plants. Environ. Pollut..

